# Quality optimisation in colonoscopy: a function of time of colonoscopy or bowel preparation

**DOI:** 10.11604/pamj.2019.32.205.16016

**Published:** 2019-04-26

**Authors:** Haytham Abudeeb, Khurram Khan, Min Maung, Lee Malcomson, Alistair Brown

**Affiliations:** 1 Hairmyres Hospital, General Surgery, Colorectal Department, Glasgow, United Kingdom; 2 Christie Hospital, Colorectal & PTS Department, Manchester, United Kingdom

**Keywords:** Colonoscopy, bowel preparation, poor bowel preparation, incomplete

## Abstract

To test the hypothesis claimed in recent studies that quality of bowel preparation for colonoscopy could be influenced by the time of the day colonoscopy is performed. Do patients in morning list have better bowel preparation than those on the afternoon list? Retrospective analysis of 736 consecutive patients who had colonoscopy from 1^st^ August to 31^st^ December 2012. Patients with poor bowel preparation (Boston Bowel Prep Score 6 or less) were identified (n = 242). Colonoscopy reports of these patients analysed. Patients were stratified into two groups (am and pm) and results compared. Mean patient age 63.9 years (range 19-89). Male to female ratio 1:1. 92% of patients were given Moviprep. for bowel preparation. 32.9% (242/736) of patients were identified as having inadequate bowel preparation. 37.7% of morning list patients had poor bowel preparation. 26.7% of afternoon list patients had poor bowel preparation. 14.7% (108/736) had incomplete colonoscopy, of which 26.9% (29/108) were due to poor bowel preparation. The commonest reasons for incomplete examination were patient discomfort & bowel looping. Our study demonstrates that morning session patients had poorer bowel preparation than the afternoon session patients in contrast to published evidence in recent literature. This implies that timing of bowel preparation is probably more important than timing of colonoscopy. Poor bowel preparation does not seem to have a significant impact on the colonoscopy failure rate in this series.

## Introduction

Colonoscopy remains the gold standard investigation for colorectal disease and plays an important role in both diagnosis and therapy. Since the introduction of Colorectal screening and polyps' surveillance, much effort, skills and expenses are invested in endoscopy units and training to standardize outcome, reduce complications and maintain colonoscopy as safe, effective and tolerable examination. Limitations still exists to a complete colonic examination even in the hands of experienced endoscopist with bowel preparation undoubtedly one of the most important factors that affect the completion of colonoscopy. The cecal intubation rate and adenoma detection are two of the main quality endoscopic indices, both of which are directly related to the quality of preparation [1, 2]. Inadequate bowel preparation for colonoscopy can result in missed lesions, cancelled procedures, increased procedure time, increased costs and a potential increase in adverse event rates [3-5]. Factors affecting the quality of bowel preparation are generally divided to patient related factors and procedure related factors. Patient related factors, which cannot be influenced, are increasing age, male gender, presence of comorbidity, colorectal pathology, socioeconomic status and obesity, while procedure related factors are adherence to bowel preparation instructions, timing of bowel purgative administration and appointment waiting time for colonoscopy [6]. It has been proposed that scheduling of colonoscopies in the afternoon compared to the morning may be an independent predictor of an incomplete colonoscopy and inadequate bowel preparation [7] but more studies have given more emphasis on the importance of the timing of giving the bowel preparation and on split dosing regimen [8, 9]. The aim of our study was assessing whether the quality of bowel preparation for colonoscopy can be influenced by the timing of the day the colonoscopy performed. In this paper data from a district general hospital analysed and presented with comparison between morning and afternoon colonoscopy lists made to address one question: do patients in morning list have better bowel preparation than those on afternoon list?

## Methods

This study is a retrospective observational study done at Hairmyres Hospital, District General Hospital in Glasgow, UK. Data of patients who had a colonoscopy from 1^st^ of August to 31^st^ December 2012 were collected on two stages initially 1^st^ of august till 30^th^ of September and then extended to the end of that same year. All patients with poor bowel preparation, which was identified as Boston Bowel Preparation score of 6 or less, their reports were collected and analysed through case notes search and stratified to AM (9:00 to 12:30) group and PM (1:30 to 5:00) group. All colonoscopies were performed by trained endoscopist or under supervision of endoscopy trainer. Bowel preparation were given the day before the procedure in split dose regimen with first dose given at 5pm and second dose given at 10pm. Timing for the bowel preparation was the same for both morning and afternoon patients but some afternoon patients were given an extra dose of phosphate enema in the morning of the procedure. All patients were kept on fluids only the day before and patients who had previous failed colonoscopy, due to poor bowel preparation, were kept on low residue diet for two weeks prior to the colonoscopy. Patient's colonoscopies reports were analysed and results, in terms of bowel preparation, were compared as a primary outcome. Data collected from patient's records retrospectively after identification of all endoscopy lists during that period and multiple further factors collected including age, gender, indication, incompletion cause, endoscopist, and any further tests performed and were recorded as secondary outcomes. Statistical analysis was done using two sample t-test (P<0.001).

## Results

**Figure 1:**
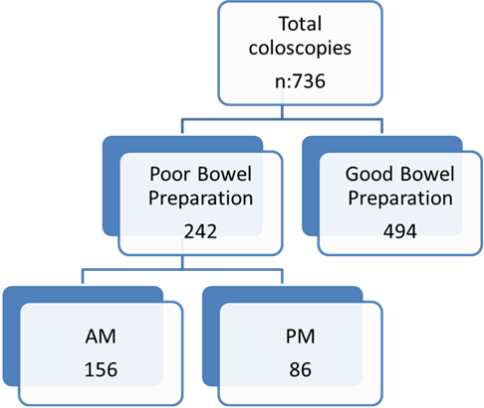
Flow chart

**Figure 2:**
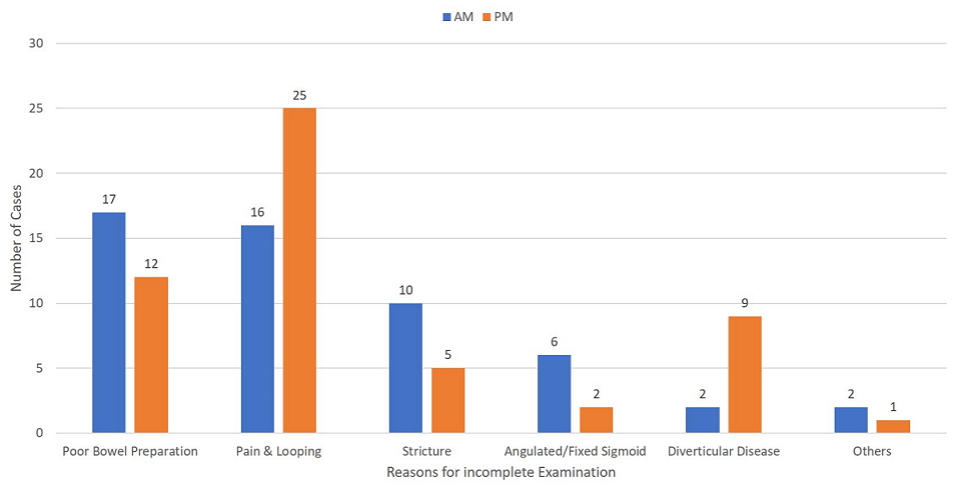
Reasons for incomplete examinations

Seven hundred and thirty six patients had colonoscopy during the period 1^st^ of August 2012 to 31^st^ of December 2012 (Figure 1). Four hundred and fourteenth patients had their colonoscopy performed during a morning session (9:00 to 12:30) and three hundred and twenty two patients had their colonoscopy performed during an afternoon session (13:30 to 17:00). Mean patient age was 63.9 years (range 19-89). Male to female ratio 1:1. 92% of patients were given MoviPrep for bowel preparation. 32.9% (242/736) of patients were identified as having inadequate bowel preparation. 14.7% (108/736) had incomplete colonoscopy, of which 26.9% (29/108) were due to poor bowel preparation (Figure 2).

**Figure 3:**
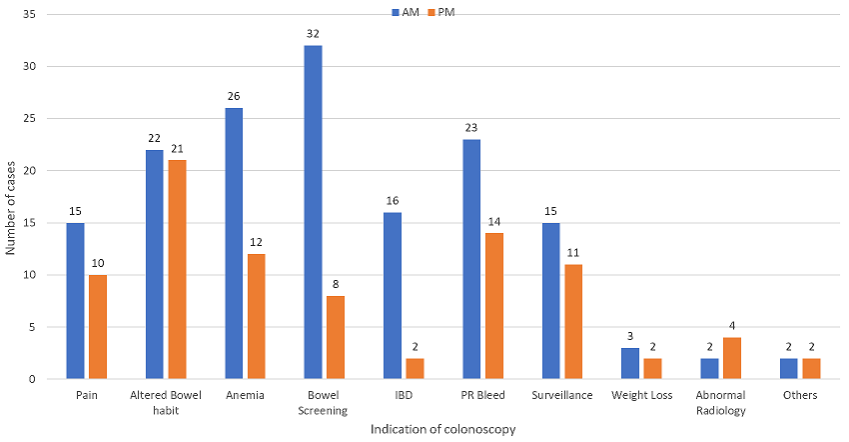
Indications of colonoscopy in patients with poor bowel preparation

**Morning lists:** The morning group (n: 414) had 156 (37.7%) patients with poor bowel preparation which is Boston bowel prep. score of 6 or less. Mean age is 62.9 years (19-89). 148 patients with poor bowel preparation had MoviPrep for bowel preparation with 3 using Kleen prep., 2 patients using Picolax, 2 patients used enema only and one patient had no bowel preparation. Of the poor bowel preparation colonoscopies, 109 procedures were performed by a consultant, 16 by trainee and 31 were completed by specialist nurse. Indications for the colonoscopies with poor bowel preparation ranged from bowel screening (32), anemia (26), PR bleed (23), altered bowel habit (22), IBD assessment (16), surveillance (15), Pain (15), weight loss (3), abnormal radiology (2), faecal incontinence (1) and abdominal mass (1) (Figure 3). The male to female ratio in AM group with poor bowel preparation was 1.3:1 with 88 males to 68 females. Incomplete examination in the AM group was 34.6%(54) with only 17 patients due to poor bowel preparation while other causes of incompletion were discomfort and looping (16), stricture (10), angulated/fixed sigmoid (6), diverticular disease (2), unable to retain air (1) and severe colitis (1) (Figure 2). 14 patients were re-scoped and 25 patients had further radiological assessments.

**Afternoon lists:** The afternoon had 86 (26.7%) poor bowel preparation with mean age 64.9 (31-86) and 32 males to 54 females with M: F ratio of 1:1.7. Bowel preparation was again predominately MoviPrep. (83), with 3 patients using Picolax. 38 procedures with poor bowel preparation was performed by a consultant, 17 by a trainee and 31 were performed by specialist nurse. Indication in the PM for colonoscopies with poor bowel preparation group ranged from altered bowel habit (21), PR bleed (14), Anemia (12), surveillance (11), pain (10), bowel screening (8), abnormal radiology (4), IBD assessment (2), weight loss (2) and faecal incontinence (2) (Figure 3). Incomplete examination in the PM group were 54 patients (62.97%) of which 12 were due to poor bowel preparation while other causes were discomfort and looping (25), diverticular disease (9), strictures (5), angulated/fixed sigmoid (2) and abdominal hernia (1) (Figure 2). 11 patients were re-scoped and 29 went on to have further radiological assessments. 736 patients had colonoscopy during the period between 1^st^ of august and 31^st^ of December 2012 of which 242(32.9%) patients had poor bowel preparation. 156 (38.4%) patients group (n: 322) were in morning lists with 86 (26.4%) patients were in the afternoon lists shown a better bowel preparation in the afternoon list patients (p<0.001).

## Discussion

Contrary to the recently published literature, this study has shown that patients had better bowel preparation in the afternoon lists in comparison to the patients in the morning lists. This only establishes the association of timing of bowel preparation administration and timing of colonoscopy. Many studies have shown evidence to support the split dose regimen for bowel preparation compared to single dose bowel preparation given the day before [8, 9]. Ideal timing of the bowel preparation, for the day before bowel preparation, would be 5 and 10 pm doses [10, 11] and this was followed by our endoscopy unit with addition of phosphate enema for the afternoon some patients in the morning of the list. Most of our patients used MoviPrep for bowel preparation, approximately 95%, with small group of patients had other preparation which included picolax and klean preparation. No obvious evidence noted in favour of any of the preparations in the study and it is well known from published literature such as Belsey *et al* that both polyethylene glycol electrolyte solutions and sodium phosphate are equally effective [11, 12]. Our patients were kept on clear fluids only the day before their procedure. No special diet requirements are included in our instructions but patients who had a failed colonoscopy due to poor bowel preparation were advised to be on low residue diet prior to their second colonoscopy at least for 2 weeks. The effect of diet before colonoscopy is not completely clarified and there is no clear evidence to support any dietary restrictions but it is acknowledged that low residue diet has improved patient satisfaction and the quality of the bowel preparation during the procedures [11]. We have made no distinction between our morning and afternoon patients in the timing of the administration of bowel preparation and diet and fluids given, but we have add an extra phosphate enema in the morning of the list for some of the afternoon list.

Interestingly our study has shown that poor bowel preparation did not seem to have a significant impact on the completion rate for our colonoscopies, in fact our completion rate for the morning lists were better than the afternoon with approximately 13% failure rate in the morning and only 31.5% of those are due to poor bowel preparation. Our completion rate in the afternoon was 83.2% with failure rate of 16.8% and 22% of those are due to poor bowel preparation. This is again contrary to published data that poor bowel preparation reduced the cecal intubation and effect completion rates by up 10% which is the recognised national figure [13]. Although caecum had been reached in these examinations, it would be difficult to accept this as complete examination as polyp detection rates are reduced in presence of poor bowel preparation and repeat colonoscopy is required for further assessment. Clear instructions are given to all patients with colonoscopy information leaflets, but unfortunately little information available on patients' compliance. It was noted from endoscopy unit staff that some patients found it difficult completing the required volume for the MoviPrep. Other patients also mentioned that the adherence to the timing was challenging due to the volume which added further discrepancy to the starting and finishing time between our patients. Patients’ heterogeneity was another limiting factor. Age and male gender both considerate independent predictive factors for inadequate bowel preparation [6] and in our study male to female ratio was 1:1, in the poor bowel preparation group, with an age range from 19 to 89 years old. This further complicated but the vast range of co morbidities associated with our patients which included diabetes, abdominal surgeries, multiple medications and different socioeconomic status which are negative predictive factors and increase the risk of confounding. Inflammatory bowel disease is again an independent predictive factors of poor bowel preparation, which was not well presented in both group. In fact more patients with IBD were colonoscoped in morning list.

## Conclusion

It is imperative that we recognise the most appropriate timing for the administration of our bowel preparation regardless of the type used as it seems that timing of purgatives is the most important factor in bowel preparation. The day before bowel preparation is still an acceptable option, but with trends to split dose regimen and same day bowel preparation for afternoon lists. Endoscopy lists will continue to be performed throughout the day with various outcomes but bowel preparation should have no effect if given appropriately. What is important is how bowel preparations are used, when to administrate the preparation and to what extent our patients will adhere to the instructions given. Multi-centre randomised control trial is still required with mutivariate analysis to provide the ideal bowel preparation regimen that can be standarised, which will include the appropriate bowel Preparation, timing of administration and dietary restrictions if any required.

### What is known about this topic

- Split dose bowel preparation the day before is the most commonly used regimen for bowel preparation in the UK;
- Morning colonoscopy patients have better bowel preparation in comparison to patients on afternoon lists;
- Poor bowel preparation has a significant impact on colonoscopy failure rate.

### What this study adds

- Split dose the day before bowel preparation is still an appropriate option with consideration of same day bowel preparation for afternoon lists;
- Morning list patients had poorer bowel preparation in this series indicating timing of purgatives is more important than timing of the list;
- Caecal intubation are not affected by bowel preparation in this series, but colonoscopic examination might not be adequate.

## Competing interests

The authors declare no competing interests.
